# Epinephrine, Pregabalin, and Crizotinib as Three Medicines with Polish Implications over Three Last Centuries and in View of Three Different Drug Discovery Approaches

**DOI:** 10.3390/biomedicines12092021

**Published:** 2024-09-04

**Authors:** Piotr Kawczak, Igor Feszak, Tomasz Bączek

**Affiliations:** 1 Department of Pharmaceutical Chemistry, Faculty of Pharmacy, Medical University of Gdańsk, 80-416 Gdańsk, Poland; tomasz.baczek@gumed.edu.pl; 2 Institute of Health Sciences, Pomeranian University in Słupsk, 76-200 Słupsk, Poland; igorfeszak@gmail.com; 3 Department of Nursing and Medical Rescue, Institute of Health Sciences, Pomeranian University in Słupsk, 76-200 Słupsk, Poland

**Keywords:** epinephrine, pregabalin, crizotinib, drug discovery, history of medicine

## Abstract

The discovery of epinephrine (adrenaline) and its subsequent implications in medicine owes significant contributions to Cybulski across different centuries, who, in 1894, was pivotal in identifying the adrenal medulla’s role in blood pressure regulation and naming the active substance “*nadnerczyna*”, known today as adrenaline. His work demonstrated the adrenal glands’ critical function in the body’s regulatory mechanisms beyond the nervous system. Cybulski’s groundbreaking research laid foundational knowledge for future endocrinological studies and pharmaceutical advancements. In the late 20th century, Andruszkiewicz collaborated with Silverman at Northwestern University to develop pregabalin, the active ingredient in Lyrica. Their innovative synthesis of gamma-aminobutyric acid derivatives led to a significant advancement in treating epilepsy, neuropathic pain, and fibromyalgia. Andruszkiewicz’s expertise in organic chemistry and enzymology was crucial in this collaborative effort, resulting in the successful development and commercialization of Lyrica. Additionally, Mroczkowski’s leadership at Pfizer contributed to the development of crizotinib, a notable anaplastic lymphoma kinase and proto-oncogene 1 tyrosine-protein kinase inhibitor used to treat specific types of non-small cell lung cancer. Her work exemplifies the continuing influence of Polish researchers in pioneering drug discovery and advancing therapeutic treatments over the past three centuries. These contributions highlight Poland’s significant role in global pharmaceutical innovations and medical research.

## 1. Introduction

The study of catecholamines plays a significant role in the history of physiology, biochemistry, and pharmacology. Epinephrine, also known as adrenaline, was the first hormone to be isolated from an endocrine gland and purified, even before the term “hormone” was introduced. It was also the first hormone to have its structure and biosynthesis fully elucidated [1]. Epinephrine is a crucial hormone produced by the body during stressful situations, primarily functioning to prepare the body for a swift reaction, often known as the fight-or-flight response [2]. It impacts multiple body systems, elevating heart rate, blood pressure, and blood sugar levels, thereby enhancing our capacity to respond rapidly to urgent situations [2,3]. This potent hormone, released in moments of intense stress or danger, initiates a cascade of reactions that ready the body for immediate action [2,4]. Epinephrine is frequently used across multiple settings due to its dual role as both a medication [5,6] and a hormone [7,8].

Gabapentinoids are GABA analogs, but they do not directly interact with GABAergic systems and do not bind to GABA or benzodiazepine receptors. Instead, their primary mechanism involves binding to the α2δ (1 and 2) subunits of voltage-dependent calcium channels. Initially introduced for epilepsy treatment, gabapentinoids have since been approved for various other medical conditions due to their effectiveness [9,10]. Pregabalin, chemically known as (S)-3-(aminomethyl)-5-methylhexanoic acid, is the pharmacologically active S-enantiomer of a racemic 3-isobutyl gamma-aminobutyric acid analogue. It is widely recognized for its anticonvulsant and analgesic properties [11,12,13]. Structurally similar to gabapentin, pregabalin targets the alpha2-delta protein, a component of voltage-gated calcium channels. By binding to these subunits, pregabalin reduces the release of certain neurotransmitters, which can help lower neuronal excitability and manage seizures [14,15].

Crizotinib is a type of cancer growth blocker characterized by its aminopyridine structure and functions as a protein kinase inhibitor by competitively binding to the ATP-binding site of specific kinases [16,17]. This oral medication inhibits multiple tyrosine kinase receptors. It is believed to impact cancer cells by affecting their growth, migration, and invasion, and may also inhibit angiogenesis in tumors. Recent progress in cancer biology and oncogene research has pinpointed several molecular targets for cancer treatment. Targeted therapies, such as crizotinib, interfere with proteins that drive tumor cell growth, resulting in reduced tumor proliferation and increased cell death [16,18].

This paper examines the key contributions to drug discovery and pharmaceutical development by exploring historical and scientific advancements, with a particular focus on the pioneering work of Cybulski, Andruszkiewicz, and Mroczkowski, highlighting their contributions to the development of epinephrine, pregabalin, and crizotinib, emphasizing the substantial influence of Polish scientific achievements on contemporary medical practices and pharmaceutical research.

## 2. Epinephrine

The ductless glands of the body, especially the small structures located above the kidneys known as the adrenal or suprarenal glands, baffled early anatomists. With no evident connections to other organs, their function and purpose remained a mystery for centuries. These glands develop early in the fetus, remain functional throughout life, and have an extensive nerve supply, suggesting they play an important role, but this was the only hint available. In 1716, the Académie des Sciences de Bordeaux held an essay competition hoping participants could explain the function of these glands, but despite creative entries, no prize was awarded. Thomas Addison was the first to link adrenal failure to a disease, establishing that the adrenals are vital for life. In 1855, he described a fatal condition marked by anemia, general languor and debility, notable weakness of the heart, stomach irritability, and a distinctive change in skin color. Investigating further, he discovered that all affected patients had diseased adrenal glands. The same year, the two zones of the adrenal gland, the cortex and medulla, were observed under a microscope, though this did not clarify their function. In 1848, German physiologist Arnold Berthold castrated a rooster and reimplanted the testes into its abdomen. The rooster’s comb continued to flourish, indicating that the testes’ function was maintained. This experiment provided crucial evidence that these ductless (endocrine) glands released chemicals directly into the bloodstream. Subsequent research focused on identifying the nature of these substances [19,20].

From 1877 to 1885, Napoleon Cybulski served as an assistant at the Institute of Physiology. In 1885, he completed his doctorate with a thesis on measuring blood flow velocity using an apparatus he created, the photohematochrometer. His research included studying the impact of the phrenic nerve on respiratory rate and analyzing the larynx and vagus nerves. In the same year, Cybulski was appointed chairman of the Institute of Physiology at Jagiellonian University in Kraków, part of the Austro-Hungarian Empire at the time. At the university, he held the positions of dean of the medical faculty and later rector. His scientific career flourished in Kraków. Cybulski made a significant contribution to the history of medicine, particularly in 1894. Together with his assistant Władysław Szymonowicz, he observed pronounced hypertonia and bradycardia following the intravenous administration of extracts from the adrenal medulla. These scientists observed that removing both adrenal glands in dogs leads to a drop in blood pressure, which can be countered by injecting an aqueous extract from the adrenal medulla. They also demonstrated that this extract raises blood pressure in animals with intact adrenal glands and that the active substance is found not only in the adrenal glands but also in the venous blood flowing from them. This extract, whose chemical composition was unknown then, was named *nadnerczyna*. Cybulski called the substance *nadnerczyna*, which in Polish literally translates to “adrenaline” (with *nadnercze* meaning “adrenal gland” in Polish). They demonstrated that the adrenal glands were responsible for its production and release into the bloodstream. Today, we know that *nadnerczyna* consists of various active substances, primarily noradrenaline and dopamine, which are metabolically transformed into adrenaline. Cybulski and Szymonowicz published their findings in the esteemed “Zentralblatt für Physiologie” in March 1895. Simultaneously, British scientists Oliver and Schäfer were researching the adrenal glands’ crucial function, achieving results consistent with those of the Kraków researchers. Oliver and Schäfer acknowledged the Polish scientists’ work, admitting that their Kraków counterparts had reached more advanced conclusions. Nevertheless, due to their discoveries, Cybulski and Szymonowicz are recognized as the pioneers in identifying adrenaline (noting that the term “adrenaline” was first introduced in 1901 by the Japanese scientist Jōkichi Takamine) [21,22,23]. Cybulski’s work was groundbreaking, not only because he identified the “hormone of fight or flight”, but also because he demonstrated that the nervous system is not solely responsible for the regulatory functions of a living organism, contrary to the prevailing belief at the time. The study’s findings were unexpected, as it was previously thought that the nervous system alone managed the body’s regulatory functions. From this point forward, Cybulski’s name will always be linked to this substance, although his scientific contributions extend far beyond this discovery [24,25,26].

In 1894, at University College London, English physician George Oliver and physiologist Edward Schafer observed the significant impact of adrenal medulla extract on the heart rate and blood pressure of animals. They used handmade laboratory equipment composed of steel hooks, fine cotton threads, pulleys, and writing pens. Their findings garnered attention within the scientific community: the extract from adrenal glands (suprarenal glands), located above the kidneys, increased heart rate and blood pressure by causing arteriole contraction. Oliver and Schafer then exposed the adrenal extract to various conditions—including heat, acid, and peptic digestion—to explore its physical and biochemical properties. Their pioneering work laid the groundwork for John Jacob Abel, an American biochemist and pharmacologist at Johns Hopkins University in Baltimore. Abel’s research culminated in the purification of the extract’s active ingredient, epinephrine, in 1899. However, the purity of Abel’s epinephrine was contested by Otto von Fürth, an Austrian physician, physiologist, and biochemist, and by Jokichi Takamine, a Japanese biochemist. Takamine, motivated by the well-recognized therapeutic potential of the suprarenal extract, succeeded in isolating a pure, stable, crystalline form of epinephrine, which he named adrenaline. In 1901, Aldrich determined its empirical formula, C_9_H_13_NO_3_, and the purified product was promptly patented by Parke-Davis & Company [27].

In 1905, British physiologists William Bayliss and Ernest Starling introduced the concept of a hormone as an internal messenger, secreted by one organ to influence the function of another. When considering the effects of epinephrine, it quickly became clear that it must be classified as a hormone. British physiologist John Langley and physician/physiologist Thomas Elliott laid the groundwork for the idea of drug receptors. Langley observed that the effects of adrenal extract were similar to the electrical stimulation of sympathetic nerves, while Elliott proposed that epinephrine might be released from sympathetic nerve terminals. Supporting this hypothesis, British chemist George Barger and English pharmacologist and physician Henry Dale found that sympathetic nerve activity, measured by the contractile response of a cat uterus in vivo, could be triggered by epinephrine. Recognizing the link between epinephrine and sympathetic nerve activity, London-based physician Brian Melland suggested that epinephrine induces bronchial muscle relaxation by targeting the vagus nerve. American physiologist Walter Cannon further developed the idea of hormones, like epinephrine, being involved in maintaining homeostasis, which eventually led to theories about internal and external signaling mechanisms and feedback responses. The therapeutic potential of epinephrine was widely acknowledged even before a detailed understanding of its mechanism of action was fully established. Manufacturers soon began developing synthetic forms of epinephrine. In 1904, German chemist Friedrich Stolz synthesized the first synthetic hormone by creating a ketone form of epinephrine, called adrenalone. Large-scale production of synthetic epinephrine became possible when Stolz converted adrenalone to adrenaline, or epinephrine, in 1906. The synthetic form of epinephrine was found to be more effective than crude adrenal extracts, which had minimal impact on disease. In 1905, American physiologist Carl Wiggers demonstrated the vasoconstrictor effects of synthetic epinephrine on cerebral blood flow [27].

The patenting of Adrenalin marked a significant shift in pharmaceutical patent law. The issues at hand were the originality and priority of Takamine’s discovery and whether it was appropriate to patent a naturally occurring substance. Judge Learned Hand ultimately ruled in favor of Takamine and Parke, Davis & Co, despite feeling unprepared to handle the complex chemical information: “I cannot stop without calling attention to the extraordinary condition of the law which makes it possible for a man without any knowledge of even the rudiments of chemistry to pass upon such questions as these.” The patent was based on the extraction technique and the product’s marketability, and this case is frequently cited in modern debates on gene patenting, demonstrating its lasting impact. Without regulatory authorities to oversee its use, adrenaline was quickly applied in many areas. It was used topically to control bleeding in the eye, nose, urethra, vagina, and stomach. Parke-Davis manuals suggested various applicators, including sprays and camel hair brushes. In 1903, Heinrich Braun, a pioneer in local anesthesia, reported that adrenaline prolonged the effect of cocaine local anesthesia. Following this, Parke-Davis began selling combinations of cocaine with adrenaline, as well as mixtures of adrenaline with synthetic local anesthetics like beta-eucaine and novocaine. Physicians found that adrenaline improved conditions like allergic rhinitis and hives, leading to further research into its use for allergies. In 1900, Solomon Solis-Cohen suggested using adrenaline tablets for asthma, believing vasoconstriction would improve airflow. Though oral administration of adrenaline proved ineffective, his work sparked additional research. By 1903, Bullowa and Kaplan successfully treated asthma with subcutaneous adrenaline injections, establishing the ineffectiveness of oral administration and recognizing adrenaline’s role in bronchodilation [28,29,30,31,32]. Adrenaline’s anti-inflammatory properties extended its use to treating gonorrhea, providing symptom relief, though claims of curing bubonic plague, bedwetting, and enabling gender selection before conception were less credible. Even respected institutions made bold claims; during the 1916 polio epidemic, Samuel Meltzer at the Rockefeller Institute experimented with injecting adrenaline into the cerebrospinal fluid of affected children, though no benefits were proven. This controversial treatment was occasionally used into the 1930s. The most crucial application of adrenaline was discovered by surgeon George Crile. Through controlled experiments, he documented the nature of surgical shock and proved in 1903 that adrenaline was effective in its treatment, revolutionizing surgical shock management. Crile also found that injected adrenaline could revive patients from cardiac arrest. Within a few years of its discovery, adrenaline had become an essential drug [33,34,35,36].

Bodon was not the first to propose using adrenaline for anaphylaxis treatment, but after his 1923 publication, the use of adrenaline for this condition significantly increased, particularly via intracardiac and intravenous routes. Adrenaline’s bronchodilatory properties were identified in 1903, and by the 1920s, it was widely used to treat asthma. Patients often self-administered it subcutaneously during acute attacks. With advancements in medical equipment making intravenous administration more feasible, it was found to be more effective for status asthmaticus. For milder asthma attacks, subcutaneous injections were eventually replaced by inhaled adrenaline. James Graeser and Albert Rowe were pioneers in suggesting oral inhalations of adrenaline. They used a 1 in 100 solution and showed it was more effective and had fewer side effects than injections for many patients. Consequently, several companies began producing specialized formulations for commercial atomizers. These solutions were more concentrated than intravenous ones, raising new safety concerns. The release of Burroughs Wellcome and Co’s 1% vaporole led to a British Medical Journal editorial emphasizing the need for prominent warnings: “The bottles of vaporole solution and their containers are labelled ‘Caution—Not for injection,’ but we would suggest the advantage of making this warning somewhat more prominent.” This highlighted the potential for errors even in the early days of intravenous medication. By the 1950s, adrenaline had become a well-established treatment for many life-threatening conditions. Despite the development of other sympathomimetic amines, it remains a vital component of all resuscitation protocols [28,35,37,38].

**Figure 1:**
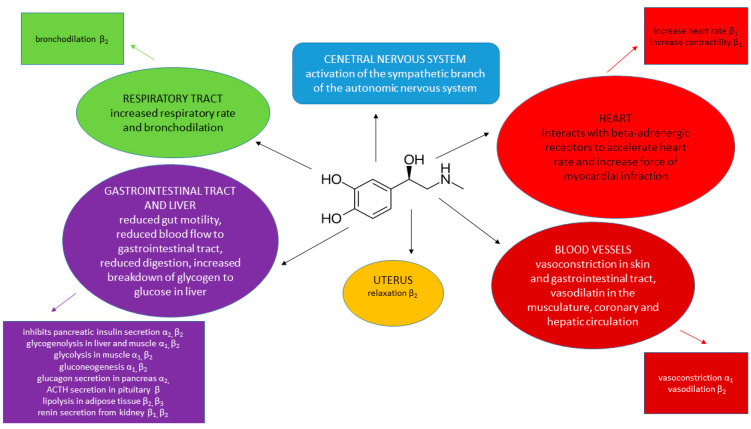
Major physiological effects of epinephrine based on Ref. [39].

The major physiological effects of epinephrine on the target organ/tissue together with the receptor type, are presented in Figure 1. In addition, Table 1 presents associated conditions of epinephrine use, Table 2 presents side effects of epinephrine, and Table 3 presents drug interactions for epinephrine.

## 3. Pregabalin

Gabapentin and pregabalin are synthetic derivatives of γ-aminobutyric acid (GABA), sharing similar biological activity but possessing increased lipophobicity, which enhances their effectiveness as anticonvulsant medications. This increased lipophobicity is due to additional alkyl groups on gabapentin and pregabalin compared to natural GABA. Gabapentin serves as the active ingredient in neurontin, while pregabalin, also known as 3-alkyl γ-aminobutyric acid, is the primary component of Lyrica. The creation of both gabapentin and pregabalin originated from researchers investigating the basic mechanisms that lead to the development of epilepsy [79,80].

Pregabalin, marketed solely under the brand name Lyrica until 2019, is a medication with anticonvulsant, analgesic, and anxiolytic properties, used to treat epilepsy, neuropathic pain, fibromyalgia, opioid withdrawal, and generalized anxiety disorder [79,81,82,83].

The International League Against Epilepsy (ILAE) defines epilepsy as a brain disorder characterized by at least two unprovoked seizures occurring more than twenty-four hours apart. This condition impacts over fifty million individuals globally, with more than 80% of cases found in developing countries [79,84,85].

The pursuit of anti-epileptic drugs (AEDs) commenced in the 19th century, but it was only possible after epilepsy ceased to be viewed as a “sacred disease” curable solely by divine intervention, and societal discrimination against those affected had diminished [79,86].

The International Association for the Study of Pain (IASP) defines neuropathic pain as pain caused by a lesion or disease impacting the somatosensory nervous system. It is estimated that chronic pain with neuropathic traits affects 7–10% of the general population [79,87,88,89].

Gabapentin and pregabalin have been found to bind to voltage-gated calcium channels at the α2-δ subunit, leading to alterations in neurotransmitter release. Both drugs have demonstrated effectiveness compared to placebo treatments in individuals suffering from various neuropathic pain conditions [79,90].

The development of Lyrica unfolded in three distinct phases. The first phase, from 1988 to 1989, involved synthesizing and studying pregabalin at Northwestern University. In 1990, Northwestern’s Technology Transfer Office licensed the chemical composition to Parke-Davis, which conducted animal pharmacokinetic and metabolism experiments for six months, followed by two years of animal toxicology studies. The second phase consisted of clinical trials, starting in 1995 after filing an Investigational New Drug Application (IND), and these trials spanned over eight years. The final phase was the Food and Drug Administration (FDA) approval in late 2004, which allowed Lyrica to be introduced to the market. In summary, the development of Lyrica was a lengthy and intricate process, requiring multiple stages of testing and refinement [79].

The early development of Lyrica started with the collaboration between Ryszard Andruszkiewicz and Richard Silverman. Andruszkiewicz, a skilled chemist from the Gdańsk University of Technology, had a background in synthesizing enzyme inhibitors, demonstrated by his publications on glucosamine synthetase inhibitors. He joined Silverman at Northwestern University in 1988 as a visiting professor [79,91].

The discovery of Lyrica emerged from Silverman’s astute scientific intuition coupled with Andruszkiewicz’s persistent laboratory investigations. Pregabalin, the active ingredient in Lyrica, was one of the 3-alkyl GABA derivatives that Silverman assigned Andruszkiewicz to synthesize in 1988. Silverman was intrigued by these compounds’ potential to treat epilepsy based on two hypotheses. First, he speculated that adding carbon atoms could enhance the compounds’ ability to penetrate the blood–brain barrier, often improving their lipophilicity. Second, he hypothesized that generating various alkyl analogs might yield a compound that selectively inhibited GABA transaminase (GABA-AT) without affecting glutamate decarboxylase (GAD). Silverman reasoned that a compound possessing both traits—penetration of the blood–brain barrier and selective inhibition of GABA-AT—could effectively enhance GABA levels in the brain, potentially treating epilepsy. Andruszkiewicz successfully synthesized this series of GABA derivatives and published the findings in the journal Synthesis in 1989, with funding from the NIH. He then evaluated the synthesized molecules’ activity on enzymes extracted from pig brains and discovered that all fourteen compounds inhibited GABA-AT and activated GAD, suggesting they could enhance GABA production in the brain. These 3-alkyl GABA derivatives thus emerged as promising candidates for increasing GABA synthesis in line with Silverman’s initial hypothesis. The results were so promising that Silverman asked Andruszkiewicz to confirm them through additional testing. Subsequently, these significant findings were published in the Journal of Biological Chemistry. Silverman proceeded to send the synthesized drugs to pharmaceutical companies for further evaluation, facilitated by the university’s technology transfer office. Northwestern University’s technology transfer office (TTO) facilitated the development and market introduction of Lyrica [79,92,93].

Lyrica received approval for medical use in Europe in July 2004, initially for treating peripheral neuropathic pain and as an adjunctive therapy for partial seizures in epilepsy patients. Subsequently, it was approved by the FDA in December 2004 for managing neuropathic pain linked to diabetic peripheral neuropathy and post-herpetic neuralgia and in June 2005 for adjunctive therapy in partial-onset seizures. Finally, in June 2007, Lyrica gained FDA approval for treating fibromyalgia [79,94,95,96].

Interestingly, the mechanism of action of pregabalin turned out to be quite different from its original hypothesis. Initially, Silverman and Andruskiewicz aimed to enhance GABA levels by inhibiting GABA-AT and activating GAD. However, subsequent studies conducted by Parke-Davis revealed that pregabalin’s anticonvulsant effects are not significantly related to GAD activation or GABA-AT inhibition. Later research indicated that both gabapentin and pregabalin bind to calcium channels, reducing calcium influx into neurons. This action inhibits the excitatory neurotransmitter L-glutamate, which likely underlies Lyrica’s anticonvulsant effect [79,97,98].

Andruszkiewicz and Silverman played crucial roles in the development of Lyrica through their collaborative efforts in synthesizing pregabalin. Andruszkiewicz, already a lecturer at Gdańsk University of Technology, ventured to the United States to advance his career. During his tenure as a visiting scholar at Northwestern University, he partnered with Silverman to work on the drug’s synthesis. Visiting professors like Andruszkiewicz are esteemed scholars invited to collaborate with host institutions, typically funded by their home institutions, to conduct hands-on research akin to postdoctoral fellows. While Andruszkiewicz was already accomplished in his field, he sought collaborations with established professors like Silverman to gain experience as a corresponding author in publications—a role usually reserved for the professor funding the research and originating the idea. Andruszkiewicz’s expertise in enzymology and organic chemistry was pivotal in successfully synthesizing all fourteen analogs of GABA. His deep understanding of enzymes enabled him to promptly assess the molecules’ effects on GABA-AT and GAD, significantly contributing to their innovative approach. Upon returning to Poland, Andruszkiewicz’s passion for scientific exploration and his mastery in the field resulted in him acquiring lucrative Lyrica patents and assuming the role of corresponding author in new publications. Silverman, Andruszkiewicz’s mentor, also played a pivotal role in pregabalin’s development. His relentless pursuit of scientific knowledge and a stroke of serendipity led to the discovery. Understanding the significance of a molecule’s lipophilicity in crossing the blood–brain barrier, Silverman directed Andruszkiewicz to synthesize alkyl-substituted GABA analogs to enhance this property. He also theorized that compounds capable of both activating GABA-AT and inhibiting GAD simultaneously would effectively elevate brain GABA levels. While initial in vitro enzymatic assays supported these ideas, subsequent animal studies revealed a completely different mechanism of action for these analogs in the brain [79,97,99].

**Figure 2:**
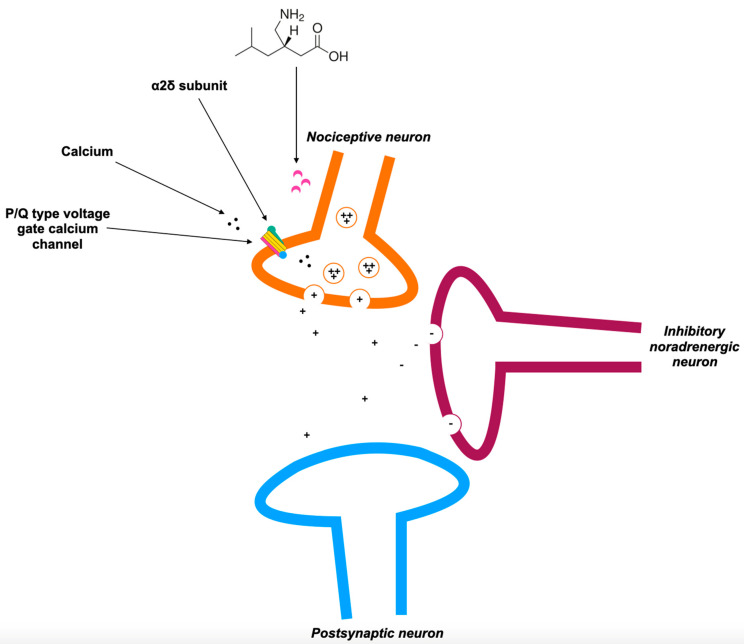
Mechanism of action of pregabalin based on Ref. [100].

The mechanism of action of pregabalin is presented in Figure 2. Furthermore, Table 4 presents associated conditions of pregabalin use, and Table 5 presents drug interactions for pregabalin.

## 4. Crizotinib

Receptor tyrosine kinases (RTKs) are essential for numerous cellular functions, including cell proliferation, migration, metabolism, differentiation, and survival. In normal cells, RTK activity is precisely regulated. However, constitutive activation of RTKs due to point mutations, gene amplification, or rearrangements can contribute to cancer development and progression. The clinical success of small-molecule tyrosine kinase inhibitors has validated this therapeutic approach, establishing several pathogenic tyrosine kinases as effective targets for cancer treatment. Examples include imatinib for gastrointestinal stromal tumors with mutant c-KIT kinase or chronic myelogenous leukemia with BCR-ABL gene translocations, erlotinib for non-small-cell lung cancer (NSCLC) with mutant epidermal growth factor receptor (EGFR), and sunitinib targeting the von Hippel–Lindau tumor suppressor (VHL)-dependent vascular endothelial growth factor (VEGF) pathway in renal cell carcinoma [106,107,108]. The receptor tyrosine kinase, mesenchymal epithelial transition growth factor c-MET, also known as hepatocyte growth factor receptor (HGFR), along with its natural ligand hepatocyte growth factor (HGF) or scatter factor, plays a crucial role in normal development, organogenesis, and homeostasis. Upon activation by HGF, c-MET triggers an invasive program that includes cell proliferation, migration, invasion, survival, and branching morphogenesis. Aberrant c-MET signaling, due to constitutive activation, gene amplification, or mutations, is present in nearly all types of solid tumors and is linked to the dysregulation of various oncogenic processes such as mitogenesis, survival, angiogenesis, invasive growth, and metastasis. Overexpression of c-MET and HGF is associated with poor prognosis or metastatic progression in several major cancers. Consequently, c-MET and HGF have emerged as prominent targets for molecularly targeted cancer therapies [109,110,111].

Anaplastic lymphoma kinase (ALK) was first identified as a potential cancer drug target 15 years prior, when it was found as a fusion kinase with nucleophosmin in anaplastic large cell lymphoma. ALK was recognized as a molecular target in NSCLC in 2007, when Dr. Hiroyuki Mano and colleagues reported that 6.7% of Japanese NSCLC patients had a fusion of EML4 with the intracellular kinase domain of ALK. In vitro studies with an ALK inhibitor, WHI-P154, and a Ba/F3 cell line model led Dr. Mano’s team to propose that ALK could be a therapeutic target in ALK-positive NSCLC. A year before Dr. Mano’s discovery, crizotinib, a small-molecule inhibitor of ALK, had entered clinical trials. Initially developed to target c-MET, crizotinib was also known to inhibit ALK. To identify ALK rearrangements as a potential target in NSCLC and determine crizotinib sensitivity, a diagnostic assay was needed. Dr. John Iafrate at Massachusetts General Hospital developed this assay using commercially available ALK fluorescence in situ hybridization (FISH) probes. This assay enabled researchers to screen NSCLC tumors, identifying key clinicopathological features associated with ALK rearrangement and screening clinically enriched populations. The first patient with advanced ALK-positive NSCLC was treated with crizotinib at the end of December 2007, just four months after the pivotal Nature publication. This patient showed an almost immediate improvement in disease-related symptoms, prompting large-scale screening efforts and the recruitment of additional ALK-positive patients at phase 1 study sites worldwide [112].

Dr. Barbara Mroczkowski served as Group Leader and Director at Pfizer La Jolla, where she guided a successful team of scientists in bringing several targeted agents to the clinical stage, many of which achieved FDA approval. At Agouron Pharmaceuticals, she contributed to solving the first high-resolution crystallographic structure of the VEGF receptors (VEGFR) kinase domain and played a pivotal role in launching Inlyta^®^ (axitinib) and Sutent^®^, selective and non-selective small-molecule inhibitors of the VEGFR kinase domain. She was also involved in the launch of Viracept^®^, one of the first FDA-approved protease inhibitors, which remains widely used in combination with reverse transcriptase inhibitors for treating HIV infection. Additionally, she led the Pfizer team responsible for the discovery and development of Xalkori^®^ (crizotinib) [113].

Crizotinib, developed by Pfizer (PF-2341066) and marketed as Xalkori, received accelerated approval in 2011 for treating patients with ALK-positive NSCLC. In 2016, it was also approved for patients with receptor tyrosine kinase ROS proto-oncogene 1 (ROS1)-positive tumors. This latest approval followed several clinical trials of crizotinib in ROS1-rearranged NSCLC. ROS1 rearrangements, found in 1−2% of NSCLC patients, are identified as oncogenic drivers, creating a distinct molecular subgroup of NSCLCs since it is rare for ALK and ROS1 rearrangements to occur in the same tumor. Crizotinib is a powerful inhibitor of ALK, ROS1, and MET kinases, binding to the ATP binding pocket as a Type I1/2B inhibitor. The drug forms hydrogen bonds with the Met1199 and Glu1197 residues. Initially, crizotinib was developed as a MET inhibitor through a structure-based drug discovery program, demonstrating potent MET inhibition in the low-nanomolar range. Subsequently, it also showed strong activity against ALK and ROS1 compared to other kinases. In cellular assays, crizotinib exhibited potent inhibitory activity against MET, ALK, and ROS1 kinases with IC50 values in the nanomolar range across various cancer cell lines. Common side effects include vision disorders, nausea, diarrhea, vomiting, constipation, edema, elevated transaminases, and fatigue [114].

ALK gene rearrangements in tumor specimens can be identified using immunohistochemistry (IHC), reverse transcription polymerase chain reaction (RT-PCR) of cDNA, and FISH. FISH is considered the gold standard for diagnosing ALK-positive NSCLC. Commercial break-apart probes used in FISH include two differently colored probes (red and green) that flank the conserved translocation breakpoint within ALK. In non-rearranged cells, these probes overlap, creating a yellow (fused) signal. In cells with ALK rearrangement, the probes are separated, resulting in distinct red and green signals. There are also atypical rearrangement patterns that respond to crizotinib [115,116,117].

The original synthesis of crizotinib by Pfizer involves two convergent routes to produce the final compound [106,114]. The first route involves preparing the pyrazole XIIIa. Activation of 1-Boc-4-hydroxypiperidine with methanesulfonyl chloride, followed by the addition of 4-iodopyrazole, yields the piperidinyl-pyrazole scaffold, which is then transformed into boronic ester XIIIa through a Pd-catalyzed reaction with bis(pinacolate)diboron. The second synthetic route produces the 2-aminopyridine scaffold. (S)-1-(2,6-dichloro-3-fluorophenyl)ethanol, obtained through a biotransformation method from the racemic analogue, undergoes a Mitsunobu reaction with 3-hydroxynitropyridine to yield a (R)-nitropyridine derivative. Reduction of the 2-nitro group followed by bromination of the heterocycle’s 5-position results in the pyridine scaffold XIIIb [118,119,120]. The final step is a Pd-catalyzed Suzuki coupling reaction of XIIIa and XIIIb, followed by Boc-deprotection to obtain crizotinib. Alternative methods have been developed to prepare 4-(4-iodo-1H-pyrazol-1-yl)piperidine and (S)-1-(2,6-dichloro-3-fluorophenyl)ethanol, which are key intermediates in crizotinib’s synthesis [121,122,123,124].

Crizotinib is a small molecule inhibitor targeting receptor tyrosine kinases such as ALK, hepatocyte growth factor receptor (HGFR, c-Met), and Recepteur d’Origine Nantais (RON). Gene translocations can modify the ALK gene, resulting in oncogenic fusion proteins that disrupt normal gene expression and signaling, promoting increased cell growth and survival in tumors. Crizotinib effectively inhibits ALK and c-Met phosphorylation in a concentration-dependent manner in cell-based assays using tumor cell lines. It also demonstrates antitumor activity in mice with tumor xenografts expressing Echinoderm microtubule-associated protein-like 4 (EML4) or nucleophosmin-anaplastic lymphoma kinase (NPM-ALK) fusion proteins or c-Met [106,112]. Initially developed to inhibit c-MET, crizotinib also potently inhibits ALK phosphorylation and signal transduction. This inhibition causes G1/S phase cell cycle arrest and triggers apoptosis in ALK-positive cells both in vitro (in laboratory settings) and in vivo (in living organisms). Following positive results from the PROFILE 1007 study, crizotinib received full FDA approval on November 20, 2013. The European Medicines Agency (EMA) initially approved crizotinib as a second-line treatment before extending its approval to first-line use on November 24, 2015 [125,126,127,128]. Crizotinib is also approved in numerous other countries for treating patients with advanced ALK-positive NSCLC. Crizotinib was initially created to target the MET pathway. Ongoing studies are evaluating its effectiveness in NSCLC patients with MET gene amplification or mutations. Additionally, crizotinib has shown promise in treating a different molecular disorder, the ROS1 gene rearrangement, found in 1% of NSCLC patients. Future research should prioritize identifying mechanisms of drug resistance and developing strategies for treatment after disease progression [129,130,131,132].

**Figure 3:**
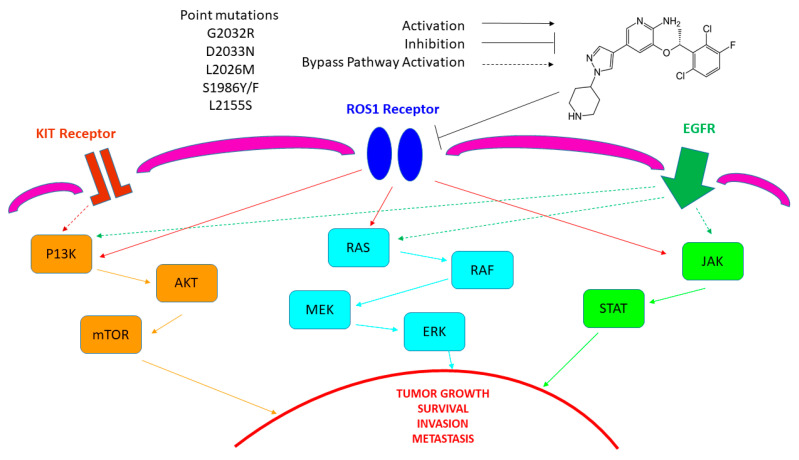
Molecular mechanisms of crizotinib based on Ref. [133].

Molecular mechanisms of crizotinib in ROS1-rearranged lung cancer patients are presented in Figure 3. Additionally, Table 6 presents associated conditions of crizotinib use, and Table 7 presents crizotinib side effects.

## 5. Conclusions

The discovery of epinephrine marked a transformative moment in understanding physiological regulation beyond the nervous system, paving the way for therapeutic applications in treating conditions like anaphylaxis and shock. This breakthrough exemplified early successes in drug discovery through natural product extraction. Pregabalin’s development showcased the efficacy of rational drug design, leveraging synthetic derivatives to enhance therapeutic potential and target specificity. Its approval in 2004 underscored the importance of structured synthesis processes in creating novel medications for diverse medical conditions. Crizotinib’s journey from a MET inhibitor to an ALK inhibitor illustrates the evolution of drug discovery methodologies, particularly in oncology. By integrating molecular biology, chemistry, and clinical research, researchers identified its efficacy against specific molecular pathways, leading to targeted therapies for ALK-positive non-small cell lung cancer. Overall, these milestones in drug discovery highlight the iterative process of scientific inquiry, from initial discovery through refinement and synthesis to clinical validation and therapeutic application. Each represents a distinct approach—natural product extraction, rational drug design, and structure-based design—that continues to shape modern medicine and treatment strategies today.

## Figures and Tables

**Table 1:** Associated conditions with the purposes of epinephrine use.

Condition	Purpose	References
Cardiac arrest	Treatment	[40,41]
Unresponsive asystole	Treatment	[40,41]
Complete heart block	Management	[42]
Unresponsive bradycardia	Treatment	[43]
Hypotension	Management in adults	[44]
Severe anaphylaxis	Treatment	[45]
Idiopathic anaphylaxis	Management	[46]
Severe hypersensitivity	Treatment	[47]
Anaphylaxis	Treatment	[48]
Angioedema	Management	[49]
Urticaria	Management	[50]
Severe asthma	Treatment	[51,52]
Mild intermittent asthma	Management	[51,52]
Uterine contraction	Management	[53]

**Table 2:** Epinephrine adverse/side effects with the mechanisms of action.

Adverse/Side Effect	Mechanism of Action	References
Cardiovascular collapse in anaphylaxis	Overactivation of β-adrenergic receptors can exacerbate cardiac dysfunction, especially in the elderly, leading to cardiovascular collapse.	[54]
Takotsubo cardiomyopathy	Rare stress-induced cardiomyopathy possibly triggered by a sudden surge in catecholamines like adrenaline.	[55]
Refractory anaphylaxis	Persistence of anaphylactic symptoms despite multiple doses of IM adrenaline due to ongoing mediator release and reduced circulating adrenaline levels.	[56]
Ventricular arrhythmias	Increased myocardial excitability due to β-adrenergic receptor activation, which may result in arrhythmias.	[57]
Myocardial ischemia	Increased myocardial oxygen demand from β-adrenergic stimulation, combined with potential coronary artery vasoconstriction from α-adrenergic effects.	[58]
Hypertension	Increased vasoconstriction through activation of α-adrenergic receptors, leading to elevated blood pressure.	[59]
Cerebral ischemia	Reduced cerebral microvascular blood flow despite increased systemic blood pressure, leading to impaired cerebral perfusion.	[60,61]
Pulmonary edema	Increased capillary hydrostatic pressure from β-adrenergic effects, causing fluid leakage into alveoli.	[62]
Tachycardia	β-adrenergic receptor stimulation increases heart rate and cardiac output.	[63]
Hyperglycemia	β-adrenergic stimulation increases glycogenolysis and gluconeogenesis, leading to elevated blood glucose levels.	[64]
Contraindication with non-selective β-blockers	Severe hypertension due to unopposed α-adrenergic receptor activation when β-receptors are blocked.	[65]

**Table 3:** Drug interactions for epinephrine with the effects of interaction.

Drug Type	Effects of Interaction	References
Tricyclic antidepressants (e.g., amitriptyline, imipramine, nortriptyline)	Increased risk of hypertension, dysrhythmia, and ectopic cardiac focus due to enhanced cardiovascular response to adrenergic stimulation.	[66,67,68]
Non-selective beta blockers (e.g., propranolol)	Marked increase in systolic and diastolic blood pressure, reflex bradycardia, and potential cardiac arrest.	[69]
General anesthetics (e.g., halothane, isoflurane, enflurane)	Increased risk of cardiac dysrhythmias during general anesthesia due to simultaneous alpha and beta receptor stimulation.	[70,71]
Cocaine	Exaggerated adrenergic response leading to hypertension, cardiac arrhythmia, myocardial infarction, and potential death.	[72]
Guanethidine (adrenergic neuron blocker)	Enhanced hypertensive response due to upregulated adrenergic receptors and competitive inhibition of neuronal reuptake.	[73]
Thyroid hormones (e.g., levothyroxine, liothyronine)	Increased risk of cardiovascular effects such as tachycardia and dysrhythmia, particularly in hyperthyroid patients.	[74]
Monoamine oxidase inhibitors (MAOIs) (e.g., phenelzine, tranylcypromine, isocarboxazid)	Risk of hypertensive crisis due to accumulation of adrenergic amines, although less frequent than previously thought.	[75,76]
Catechol-O-methyltransferase (COMT) inhibitors (e.g., entacapone, tolcapone)	Potential inhibition of the inactivation of epinephrine, leading to enhanced and prolonged adrenergic effects.	[77]
Digoxin	Increased risk of dysrhythmic activity and potential for acute myocardial infarction due to excessive adrenergic stimulation.	[78]

**Table 4:** Associated conditions of pregabalin use with the therapeutic effects according to Ref. [101].

Condition	Therapeutic Effect
Peripheral neuropathy	Significant reduction in pain in diabetic neuropathy and postherpetic neuralgia.
Epilepsy	Reduction in seizure frequency as adjunctive therapy.
Fibromyalgia	Moderate pain reduction and improvement in sleep quality.
Generalized anxiety disorder (GAD)	Significant reduction in anxiety symptoms.
Postoperative pain	Reduction in postoperative pain intensity and decreased opioid requirement.
Neuropathic pain in spinal cord injury	Reduction in pain intensity, improvement in sleep quality, reduction in anxiety and depression.
HIV-associated neuropathic pain	Lack of efficacy in pain reduction.
Central neuropathic pain (e.g., post-stroke)	Lack of efficacy in pain reduction.

**Table 5:** Drug interactions with the effects for pregabalin.

Drug Class	Effect of Interaction	References
Opioids (hydrocodone, morphine)	Increased risk of respiratory depression, opioid overdose, opioid-related deaths.	[102,103,104]
Benzodiazepines (lorazepam, diazepam, alprazolam)	Potentiation of central nervous system (CNS) depression, increased risk of sedation and respiratory depression.	[105]

**Table 6:** Associated conditions of crizotinib use with the mechanism of action.

Disease/Condition	Mechanism and Effect of Action	References
Non-small cell lung cancer (NSCLC)	Crizotinib is a tyrosine kinase inhibitor that inhibits ALK (anaplastic lymphoma kinase) expression, leading to reduced cell proliferation and increased apoptosis.	[134]
NSCLC with MET mutation	Crizotinib acts as an inhibitor of the c-MET kinase, reducing its activity, which leads to the inhibition of cancer cell proliferation.	[135]
NSCLC with ROS1 translocation	Crizotinib inhibits ROS1 kinase, leading to the suppression of cancer cell growth.	[136]

**Table 7:** Crizotinib side effects with description.

Adverse/Side Effect	Description	References
Nausea and vomiting	Common gastrointestinal side effects, observed in a significant portion of patients, often grade 1 or 2 in severity.	[134]
Diarrhea	Another frequent gastrointestinal side effect, typically mild to moderate (grade 1 or 2).	[134]
Visual disturbances	Includes delayed light adaptation and increased number of floaters, usually mild (grade 1).	[134]
Elevated transaminases (ALT/AST)	Grade 3 or 4 elevations in liver enzymes (ALT/AST) observed in a small percentage of patients, requiring dose adjustment or discontinuation.	[134]
Pneumonitis	A rare but serious side effect, occurring in less than 2% of patients, which can be fatal; requires discontinuation of the drug.	[134]
Bradycardia	Asymptomatic sinus bradycardia (heart rate ≤ 45 bpm) has been reported in some patients, usually not requiring discontinuation.	[134]
Hypogonadism	Crizotinib has been associated with rapid-onset hypogonadism in male patients, leading to low testosterone levels.	[134]
Neutropenia	Occasional grade 3 or 4 neutropenia reported, particularly in heavily pretreated patients.	[125]
Fatigue	Common side effect, which can range from mild (grade 1) to severe (grade 3).	[125]
QT interval prolongation	Rare cases of QT interval prolongation have been reported, potentially leading to cardiac arrhythmias.	[125]

## Data Availability

Data sharing is not applicable to this article.
